# Dinutuximab beta versus historical controls in the treatment of relapsed neuroblastoma: unadjusted and adjusted indirect comparisons

**DOI:** 10.3389/fonc.2025.1736165

**Published:** 2026-01-22

**Authors:** Holger N. Lode, Przemysław Holko, Aleksandra Wieczorek, Katarzyna Śladowska, Nikolai Siebert, Dominique Valteau-Couanet, Alberto Garaventa, Adela Cañete, John Anderson, Isaac Yaniv, Shifra Ash, Lucas Moreno, Juliet Gray, Roberto Luksch, Genevieve Laureys, Cormac Owens, Carla Manzitti, Sascha Troschke-Meurer, Paweł Kawalec, Ruth L. Ladenstein

**Affiliations:** 1 Department of Pediatric Hematology and Oncology, University Medicine Greifswald, Greifswald, Germany; 2 Department of Nutrition and Drug Research, Institute of Public Health, Faculty of Health Sciences, Jagiellonian University Medical College, Krakow, Poland; 3 Paediatric Haematology Oncology, Jagiellonian University Medical College, Krakow, Poland; 4 Children and Adolescent Oncology Department, Gustave Roussy, Paris-Sud University, Paris, France; 5 Oncology Unit, IRCCS, Istituto Giannina Gaslini, Genoa, Italy; 6 Paediatric Haemato-oncology Unit, Hospital Universitario y Politecnico La Fe, Valencia, Spain; 7 Developmental Biology and Cancer Department, University College London (UCL) Great Ormond Street Institute of Child Health, London, United Kingdom; 8 Schneider Children’s Medical Center of Israel, Sackler Faculty of Medicine, Tel Aviv University, Petach Tikva, Israel; 9 Department of Pediatric Hematology-Oncology, Ruth Rappaport Children’s Hospital, Rambam Health Care Campus, Technion-Israel Institute of Technology, Rappaport Faculty of Medicine, Haifa, Israel; 10 Division of Pediatric Hematology & Oncology, Vall d’Hebron Barcelona Hospital Campus, Barcelona, Spain; 11 Centre for Cancer Immunology, University of Southampton, Southampton, United Kingdom; 12 Department of Pediatric Oncology, Fondazione IRCCS Istituto Nazionale dei Tumori, Milan, Italy; 13 Department of Pediatric Hematology/Oncology and Stem Cell Transplantation University Hospital Ghent, Ghent, Belgium; 14 Department of Haemato-Oncology, Our Lady’s Children’s Hospital Ireland, Dublin, Ireland; 15 Department of Paediatrics, St. Anna Children’s Hospital, Medical University, Vienna, Austria

**Keywords:** dinutuximab beta, historical controls, indirect comparison, relapsed neuroblastoma, anti-GD2 immunotherapy

## Abstract

**Objective:**

Dinutuximab beta (dB) immunotherapy is used as maintenance treatment for relapsed/refractory neuroblastoma (NBL); however, comparative studies directly comparing dB with no dB therapy in this setting are lacking. This study aimed to indirectly compare dB (with or without interleukin-2) with no immunotherapy in patients with relapsed NBL.

**Methods:**

Three studies of dB (APN311-202, APN311-304, and APN311-303) with individual patient data, along with two historical control cohorts (INBR and R1) were included. Both unadjusted (naïve) and population-adjusted comparisons of overall survival (OS) were performed, with adjustment conducted using inverse probability or odds weighting. Harmonized inclusion criteria were applied across all study populations. The adjusted comparison used the propensity score reweighting to balance the cohorts based on key baseline prognostic factors.

**Results:**

The base-case unadjusted indirect comparison revealed that dB (with or without IL-2) significantly prolonged OS compared to historical controls not treated with dB (hazard ratio [HR], 0.43; 95% confidence interval [CI], 0.31– 0.79; p<0.001). Similarly, in the adjusted comparison, dB significantly prolonged OS compared to historical controls (HR, 0.53; 95% CI, 0.35; 0.79, p=0.002). All sensitivity unadjusted and adjusted comparisons supported the results of the base-case analysis.

**Conclusion:**

Dinutuximab beta significantly prolonged OS compared to historical control cohorts not treated with dB in both unadjusted and adjusted indirect comparisons.

## 1 Introduction

Neuroblastoma (NBL) is a malignant tumor of incompletely understood etiology, originating from neuroblasts. The peak incidence of NBL occurs during the second year of life, with 90% of cases diagnosed in children under 5 years of age (1, 2). The location of the primary tumor varies depending on the origin and migratory pathways of neuroblasts during fetal development. Most commonly, NBL arise in the retroperitoneal area of the abdominal cavity, posterior mediastinum, neck, or pelvis (3). Clinical symptoms largely depend on the location of the primary lesion. Symptoms of relapsed neuroblastoma can include among others an abdominal mass, enlarged lymph nodes in the neck, swelling and bruising of the area around the eyes, unexplained fevers, bone pain, or limping, weakness or paralysis, weight loss or poor appetite, and uncontrolled eye or leg movements (4). Additionally, symptoms may be associated with excessive secretion of catecholamines or intestinal vasoactive hormones (2). Neuroblastoma is often associated with nonspecific symptoms that may delay diagnosis, such as somnolence, lack of appetite, weight loss, abdominal pain, fever, lymphadenopathy, pallor, easy bruising, weakness, or irritability (2, 5). Relapsed disease does not need to manifest clinically as symptomatic tumor progression (6). Typically, new lesions are detected in bone or bone marrow during follow-up scanning after completion of upfront therapy with earlier detection allowing for earlier treatment initiation aiming to improve outcomes (7).

In high-risk NBL (HR-NBL), defined by metastatic disease in children older than 12 or 18 months, or by *MYCN* amplification at any age, approximately half of those who achieve remission will experience relapse (8). The median time to relapse is 13.2 months (range, 1 day to 11.4 years), and the 5-year overall survival (OS) from the time of first relapse is 20% (9). This indicates that relapse negatively affects survival, with earlier relapse associated with a higher risk of death (9). Relapsed NBL requires intensive treatment that may involve a combination of chemotherapy, immunotherapy, and other targeted therapies. The most important goal of treatment in patients with relapsed/refractory NBL is to control disease and delay progression in the short-term, with the aim of prolonging long-term disease-free survival and potentially providing a route to cure with increasing emphasis on quality of life (10).

The presence of GD2, a disialoganglioside expressed on the surface of almost all NBL cells, was identified as a target for immune recognition by anti-GD2 monoclonal antibodies (11), forming the basis for effective immunotherapy and chemoimmunotherapy. Dinutuximab beta (dB, ch14.18/CHO) is a chimeric monoclonal IgG1 antibody specifically directed against the carbohydrate moiety of GD2, which is overexpressed on NBL cells (12). *In vitro* studies have demonstrated that dB binds to GD2-expressing NBL cell lines and induces both complement-dependent and antibody-dependent cell-mediated cytotoxicity (13, 14). In the presence of human effector cells, dB was found to mediate the lysis of human NBL and melanoma cell lines in a dose-dependent manner (14). Unlike other mouse-human chimeric monoclonal IgG1 antibodies, dB is produced in Chinese hamster ovary (CHO) cells. These cells are virus free and are considered the current standard for the production of recombinant proteins used in clinical trials (15). Additionally, CHO-derived production has favorable glycosylation pattern (less amount of immunogenic Neu5Gc forms of sialic acid) (16, 17), while anti-GD2 monoclonal antibody produced in SP2/0 cells (such as dinutuximab (Unituxin^®^), contains Galactose-alpha-1,3-Galactose epitopes which have the potential to cause allergic/hypersensitivity reactions, including anaphylaxis (18–22). No Galactose-alpha-1,3-Galactose-epitopes are present on dB and the reported difference in the risk serious anaphylactic reactions is approximately two-fold in favor of dB (18), albeit head-to-head study data have not been identified. At present, dB is the only anti-GD2 treatment that has been approved by the European Medicines Agency for the treatment of NBL since May 2017 (14), and is the only anti-GD2 antibody approved for maintenance therapy both in patients with newly diagnosed and relapsed disease.

Maintenance therapy with anti-GD2 antibodies, such as dinutuximab and dB, has demonstrated efficacy in patients with HR-NBL in the first-line setting, with significant improvements in event-free survival and OS, as shown in COG and SIOPEN trials (p<0.05) (8, 11, 23). Subsequent research investigating the addition of interleukin-2 (IL-2) revealed no further benefit (8, 24, 25), leading to the recommendation that dB therapy be administered without IL-2 (26).

Furthermore, anti-GD2 immunotherapies have been established as a standard of care for the treatment of relapsed/refractory NBL when administered in combination with chemotherapy (27). Following disease control, anti-GD2 monotherapy is subsequently used as maintenance therapy, with the aim of sustaining remission and preventing further relapse (25, 28).

In a retrospective Spanish study by de las Heras et al. (2025) (29), which included patients with refractory and relapsed/progressive HR-NBL, anti-GD2 immunotherapy (administered in combination with chemotherapy, as consolidation, or as maintenance) was associated with significantly improved OS (48.4% vs. 19.4%, p=0.012), compared to no immunotherapy (29). Previously, a comparison of retrospective data to historical controls from Italian Neuroblastoma Registry (INBR) demonstrated improved overall survival on dB (p=0.002) (30). While the impact of dB on OS in patients with relapsed/refractory NBL is acknowledged, the design limitations of available studies (such as cross-over effects in prospective trials, lack of separate analyses for dB in maintenance therapy, and small sample sizes) (31, 32) preclude a reliable estimation of the effect size. No trials are currently available that directly assess dB used as maintenance therapy, either in combination with IL-2 or as a single agent, as recommended by SIOPEN, compared to no dB (i.e., no immunotherapy) in the relapsed NBL setting. Given the high unmet need among patients with relapsed NBL and the lack of alternative therapies at the maintenance stage, it is essential to conduct a reliable indirect comparison of survival outcomes. Indeed, it is likely the only viable approach to generate comparative evidence, as a parallel prospective study of dB versus non-immunotherapy control, may not be ethically feasible.

Therefore, the aim of this study was to compare OS in patients with relapsed NBL treated with dB as maintenance therapy (either in combination with IL-2 or as a single agent, as recommended by SIOPEN) with that of historical control patients who did not receive anti-GD2 immunotherapy.

## 2 Materials and methods

### 2.1 Study design and plan

This was an indirect, unanchored comparison of time-to-event data from various sources. Both unadjusted (naïve) and population-adjusted comparisons were planned, with the latter using inverse probability or odds weighting. Harmonized inclusion criteria were applied across all study populations. For the adjusted analysis, propensity score (PS) reweighting was used to balance cohorts based on key baseline prognostic factors.

### 2.2 Selection of sources

#### 2.2.1 Historical controls

Two sources of individual patient data (IPD) for historical controls were identified:

- a subset of the patient population from the study by Garaventa et al. (2009) (33), specifically relapsed patients diagnosed between 1999 and 2004 from the Italian Neuroblastoma Registry (INBR);
- patients initially treated in the control (non-dB) arm of the frontline R1 randomization of the HR-NBL-1/SIOPEN study (34) who then relapsed and became eligible for dB.

Garaventa et al. (2009) (33) analyzed data from the INBR involving children who experienced disease recurrence following treatment under protocols established by the Associazione Italiana di Ematologia e Oncologia Pediatrica (AIEOP). Treatment details are provided in Section 2 of the respective publication (33). While their original analyses were based on follow-up data censored as of December 31, 2006, the comparisons presented in this report used extended data with follow-up until December 9, 2015. Although Garaventa et al. (2009) (33) included patients diagnosed with NBL between 1979 and 2004, only those diagnosed between 1999 and 2004 were selected for this historical control comparison. This period was chosen because 1999 marked the introduction of modern multimodal induction and consolidation chemotherapy, combined with maintenance therapy using 13-cis retinoic acid (13-cis-RA; isotretinoin), which significantly improved the prognosis for patients with HR-NBL.

Garaventa et al. (2009) (33) defined relapses as the appearance of any new lesion(s) or deterioration of previous lesion(s). The timing of relapse was defined as “early relapse” or “late relapse”, using a cut-off of 18 months after achieving complete response or very good partial response.

To obtain a historical control population comparable to dB-treated patients, only individuals with relapsed NBL as their baseline disease status were selected from both data sources. Additionally, the following selection criteria were applied to the patient cohort described by Garaventa et al. (2009) (33) to ensure optimal comparability between study patients and historical controls:

- date of initial diagnosis ≥1999,
- age at initial diagnosis ≥12 months (as calculated by Garaventa et al., 2009),
- age at first relapse ≥12 months (as calculated by Garaventa et al., 2009),
- International Neuroblastoma Staging System (INSS) stage 4 at initial diagnosis or first relapse not classified as local,
- patients were alive at the auxiliary starting point (defined below).

HR-NBL-1/SIOPEN (34) is an open, multicenter randomized phase III therapy optimization study including several randomization steps. The first step, the R1 randomization phase, began in 2002 and compared 2 myeloablative therapy (MAT) regimens: busulfan and melphalan versus carboplatin, etoposide, and melphalan. The R2 randomization phase, initiated in 2009 (i.e., several years after R1), compared ch14.18/CHO (dB) immunotherapy following MAT, with or without aldesleukin (interleukin-2; IL-2), in addition to differentiation therapy with 13-cis-RA. Thus, patients enrolled in the R1 phase who received standard-of-care NBL treatment, including MAT but without immunotherapy, represent a valid historical control group for comparison with patients who received immunotherapy alongside standard treatment.

For the R1 historical control group comparison, patients from the HR-NBL-1/SIOPEN study (34) who were randomized in the R1 phase (but not in the R2 phase, as those patients received dB immunotherapy) were selected. Inclusion required a documented complete response (although HR-NBL-1/SIOPEN study enrolled patients with prior complete or partial response) following MAT, or following radiotherapy if the tumor status after MAT was unavailable, along with a recorded subsequent relapse date. Patients from the HR-NBL-1/SIOPEN study who were randomized in the R1 phase and achieved a complete response after MAT, or after radiotherapy if post-MAT tumor status was unavailable, were excluded if they experienced their first relapse prior to the completion of MAT (or radiotherapy, as applicable).

In addition to having relapsed NBL, the Garaventa et al. (2009) (33) control-selection criteria for the R1 controls were used to enhance comparability. In total, 82 patients with relapsed NBL from the historical control groups were included in the study.

#### 2.2.2 Dinutuximab beta

Six studies evaluating dB for the treatment of relapsed NBL were identified in our previous systematic review (35) and assessed for potential inclusion in the indirect comparison with historical controls (**Table 1**). Of these 6 studies, 3 (APN311–202 V1+V2 [stage I] (18, 36), and APN311–202 V3 [stage II] (18, 40), APN311-304 (37), and APN311-303 (18, 30), were included in the indirect comparison (**Table 1**), based on the following selection criteria:

**Table 1:** Studies of dB in the treatment of relapsed neuroblastoma.

Study, cohort		Comment
APN311-202 (18, 36, 40)	V1+V1 (stage I)	• Included • Prospective, 51 patients with relapsed disease
	V3 (stage II)	• Included • Prospective, 45 patients with relapsed disease (21 in experimental arm, without IL-2)
APN311-304 (37)		• Included • Prospective, 19 patients with relapsed disease • OS: long-term follow-up data available (18 patients with relapsed disease)
APN311-303 (compassionate use) (18, 30)		• Included • Retrospective, 30 patients with relapsed disease • Compassionate use (treatment or patient selection unknown and/or not representative to whole population)
Wieczorek et al., 2023 (38)		• Excluded • Only 8 patients with relapsed disease
APN311-201 (Flaadt et al., 2023) (39)		• Excluded • No IPD • Inappropriate dosing (28-day cycle) • Inappropriate treatment (haplo-SCT required as treatment of all patients)
APN311-101 (Ladenstein et al., 2013) (17)		• Excluded • Inappropriate dosing (28-day cycle; max 3 cycles; only 10 patients with recommended dose) • No IPD for relapsed patients (16 refractory)

IL-2, interleukin-2 (aldesleukin); IPD, individual patient data; OS, overall survival; SCT, stem cell transplantation.

- patient population: at least 10 patients with relapsed NBL,
- treatment: administered according to the registered protocol and clinical guidelines,
- availability of IPD.

Patients treated for relapse were identified from the selected studies. These patients had experienced at least one relapse since their initial diagnosis of NBL and had received treatment for the relapse prior to initiating immunotherapy. In total, 144 patients with relapsed NBL treated with dB (with or without IL-2) were included in the study. General information about the included dB trials is provided in **Table 2**.

**Table 2:** Key information about dB trials included in indirect treatment comparison.

	APN311-202, V1+V2 (stage I) (18, 36)	APN311-202, V3 (stage II) (18, 40)	APN311-304 (37)	APN311-303 (18)/Mueller et al., 2018 (30)
Methods	Phase I/II trial Stage I: dose schedule finding phase (V1+V2) open-label, single-arm, multicenter	Phase II trial Stage II: confirmatory phase to treat an expansion cohort (V3) – randomized controlled trial, parallel groups	Prospective, open-label, single-arm Phase II trial	Open-label, single-arm, retrospective, single-center, compassionate use
Key inclusion criteria	At study entry patients had to be > 1 year but ≤ 21 years of age. Patients >21 years but ≤ 45 years of age, fulfilling the remaining criteria, could be enrolled in the study. These patients were to be analyzed separately and were not to be included in the dose finding schedule algorithm. The purpose for the inclusion of older patients was to enable the collection of tolerability data. b) Patients had to be diagnosed with NBL according to the International Neuroblastoma Staging System (INSS) criteria. c) Patients had to have received at least 1 previous high-dose treatment followed by stem cell rescue after conventional therapy. d) Patients had to fulfill one of the following criteria: • Patients with Stage 4 NBL either: o on the current high-risk SIOPEN trial (HR-NBL-1/SIOPEN) either with primary refractory disease having had 2 or more than 2 front-line treatments or patients ineligible for the R2 randomization due to major delays after completed high-dose treatments, or standard high-risk front-line treatment (other than HR-NBL1(1.5)/SIOPEN) consisting of intensive induction, followed by high-dose treatment with stem cell rescue. • Treated and responding relapse after primary Stage 4 disease, without signs of progression at study entry; • Treated and responding disseminated NBL relapse having received autologous stem cell transplantation (ASCT) without signs of progression at study entry	Patients: 1. diagnosed with NBL according to the INSS criteria 2. aged > 1 year but ≥21 years of age at study entry (subjects >21 and ≤45 years of age could be enrolled and treated for the collection of tolerability data only) 3. who had received at least one previous high dose treatment followed by stem cell rescue after conventional therapy. 4. fulfilled one of the following • Stage 4 NBL and on the current high-risk SIOPEN trial (HR-NBL-1(1.5)/SIOPEN) either with primary refractory disease having had two or more than two front-line treatments or subjects ineligible for the R2 or respectively R4 randomization due to major delays after completed high-dose treatments or standard high-risk front-line treatment (other than HR-NBL1(1.5)/SIOPEN) consisting of intensive induction, followed by high-dose treatment with stem cell rescue • Treated and responding relapse after primary stage 4 NBL, without signs of progression at study entry; • life expectancy ≥12 weeks.	Patients aged 1–21 years with NB, according to the INSS criteria: primary refractory Stage 4 disease or had relapsed after primary Stage 4 disease, or had developed distant metastases following primarily localized NBL, and their tumor burden was controlled using conventional therapy but with measurable disease still present.	Patients at ≥ 1 year and ≤ 45 years of age at treatment start with diagnosis of HR-NBL according to the INSS criteria, i.e. INSS stage 2, 3, 4, or 4s with MYCN amplification, or INSS stage 4 without MYCN amplification or relapsed or refractory NBL of any stage. Off any standard or experimental treatments for at least 2 weeks prior to treatment start and fully recovered from the short term major toxic effects
Interventions for treatment of relapsed NBL	dB: applied from Day 8 to 18 of each 35-day cycle as a 10-day continuous infusion of 100 mg/m^2^ per cycle. IL-2: at the dose of 6x10^6^ IU/m²/day was given on Days 1–5 and Days 8–12 of each cycle. Isotretinoin: applied for 14 days starting at cycle Day 22 at the dose of 160 mg/m^2^/day	Group I: dB + isotretinoin dB LTI 5 cycles of 100 mg/m^2^ dB-LTI (days 8-17) + 160 mg/m^2^ oral isotretinoin (days 19-32) vs Group II: dB + isotretinoin + IL-2 dB LTI 5 cycles of 100 mg/m^2^ dB-LTI (days 8-17) + 160 mg/m^2^ oral isotretinoin (days 19-32) with IL-2 SC 6x10^6^IU/m^2^ (days 1-5; 8-12)	dB: 10 mg/m^2^/day as continuous infusion over the first 10 days of each 35-day cycle for up to 5 cycles (in the absence of disease progression) without IL-2 or isotretinoin. dB was planned to be administered in the hospital setting in each cycle, but if well tolerated, it could be given in an outpatient setting from day 5 of cycle 1.	dB: initially 50 mg/m^2^ in their first treatment cycle to assess feasibility and tolerability of the treatment regimen. Most patients started with SC IL-2 in the first week, followed by a combination of dB and IL-2 in the second week. The total duration of a cycle varied between 28 and 35 days. In each cycle treatment ended with oral isotretinoin after the completion of the dB infusion. A total of up to 6 cycles was given. IL-2: IL-2 was usually given SC at a dose of 6 x 10^6^ IU/m^2^/day. The majority of patient received it in two 5-day blocks (days 1–5 and 8-12). In these patients, IL-2 was given concurrently with dB on days 8-12. Initial patients, however, received IL-2 on days 1–5 only as they started with the combination of IL-2 and dB. Patients ≤ 12 kg were dosed according to body weight: 0.2x10^6^ IU/kg/day. Isotretinoin: total daily dose of 160 mg/m²/day administered in two equal oral doses twice a day for 14 days after the completion of the dB infusion. Doses were rounded to the nearest 10 mg. The starting day was either day 14 or day 21. Patients ≤12 kg were given 5.33 mg/kg/day divided into two equal doses given orally twice a day for 14 days.

dB, dinutuximab beta; HR-NBL, high-risk neuroblastoma; INSS, International Neuroblastoma Staging System; IL-2, interleukin 2; IU, international unit; LTI, long-term infusion; NBL, neuroblastoma; SC, subcutaneous.

### 2.3 Data management

The study included 144 patients with relapsed NBL treated with dB (with or without IL-2) and 82 patients with relapsed NBL from historical control groups (**Table 3**).

**Table 3:** Patient disposition in historical controls and dinutuximab beta studies.

Study	Arm	N	%, arm	%, total sample
INBR control	Historical control	34	41.46%	15.04%
R1 control	Historical control	48	58.54%	21.24%
Total		82	100.00%	36.28%
APN311-303	dB	30	20.83%	13.27%
APN311-202, V1+V2	dB	51	35.42%	22.57%
APN311-202, V3	dB	45	31.25%	19.91%
APN311-304	dB	18	12.50%	7.96%
Total		144	100.00%	63.71%

dB, dinutuximab beta.

The following information was extracted for each patient:

• patient characteristics (determined by availability for historical controls):

o date of birth (for patients from the APN311–304 study, only the year of birth was available; assumed birthday of June 30 for every patient),
o sex,
o date of initial diagnosis,
o date of relapse,
o date of immunotherapy initiation (first administration of dB),
o MYCN status (amplified, not amplified, or missing),
o INSS stage at initial diagnosis (specific stage or missing),

• outcomes (OS):

o censor date (date of the last examination or contact),
o date of death,
o event classifier (event, 1; no event – censored, 0).

### 2.4 Statistical analysis

#### 2.4.1 Starting point

The starting point for OS analyses for dB studies was defined as the initiation of dB treatment (the day the first dose was administered, irrespective of IL-2 treatment). For historical control patients, who had not received dB, an auxiliary starting point was estimated using the date of the last documented relapse.

In the base-case analysis, the starting point was estimated by adding 269 days to the date of relapse, corresponding to the median time between the first relapse and the initiation of dB and/or IL-2 treatment among patients in the APN311–202 study (contributing majority of patients to pooled dataset).

In the sensitivity analysis, the starting point was estimated using a generalized linear model (with a gamma distribution, log link, and robust variance) fitted to data on the time from relapse to initiation of dB treatment in patients from the dB arm (N = 142). The model was then used to predict the auxiliary starting point in historical control patients according to their characteristics (MYCN status, INSS stage, sex, age at initial diagnosis, and time from diagnosis to relapse).

The auxiliary starting point led to the additional selection criterion for historical control patients. The study excluded patients from historical control groups who died before the auxiliary starting point (no patient in the base-case analysis; 10 patients in the sensitivity analysis using the generalized linear model).

#### 2.4.2 Comparison of patient characteristics

The χ^2^ test was used for comparing categorical variables, while the Wilcoxon rank-sum test (or for PS-reweighted patient groups, the test for the linear regression coefficient for the study arm) was applied for continuous variables to compare patient characteristics between treatment arms.

#### 2.4.3 Unadjusted comparison of OS

An unadjusted comparison of OS was conducted using the Kaplan-Meier analysis and a simple Cox model, with study group as the only covariate, employing robust variance estimators.

Sensitivity analyses were conducted for the following scenarios:

- dB without IL-2 treatment,
- selection of dB study (each individual dB study vs the combined historical control group),
- selection of historical control (dB studies combined vs each individual historical control),
- inclusion of dB patients diagnosed up to 2009,
- patient characteristics (subgroups analyzed):

o age at the starting point (4 age groups analyzed: <2 years, 2–5 years, 5–10 years, ≥10 years),
o time from diagnosis to the starting point (reflecting the interval between age at diagnosis and age at the starting point; terciles of total sample: below 2.3 years, 2.3 to 3.7 years, 3.7 years and more),
o age at diagnosis (2 groups: <5 years, ≥5 years),
o sex (male, female),
o MYCN status (3 groups analyzed separately: MYCN amplified, MYCN non-amplified, any known MYCN status – excluding patients without MYCN status available),
o INSS stage (2 groups analyzed separately: stage 4 only, any stage excluding patients without INSS data available).

#### 2.4.4 Adjusted comparison of OS: inverse probability or odds weighting

A population-adjusted comparison of OS with population adjustment using inverse probability or odds weighting was planned. PS reweighting was used for adjusted comparison to balance key baseline prognostic factors between groups (41–43). The PS model included all baseline covariates with established or plausible prognostic impact on overall survival after relapse that were available across all cohorts. The following potential prognostic factors were considered in the PS assessment:

- time from diagnosis to the starting point (reflecting the interval between age at diagnosis and age at the starting point),
- age at diagnosis,
- sex (male, female),
- MYCN status (amplified, not amplified, missing),
- INSS stage (stage 4 or other).

Other clinically relevant factors (e.g., end-of-induction response, detailed histology, ALK status, single versus tandem transplant, duration of initial therapy, and the chemotherapy backbone used at relapse) were not collected in a harmonized way in the historical cohorts and therefore could not be incorporated into the PS model. For several of these, published evidence relates mainly to survival from diagnosis, and a persistent ‘carry-over’ or legacy effect on overall survival after relapse, beyond time to relapse, age, stage and MYCN, has not been clearly demonstrated. We did not apply multiple imputation methods because several clinically important covariates (e.g. end-of-induction response, histology, ALK status, number of frontline anti-GD2 cycles, relapse regimen) were structurally missing in the historical cohorts and thus ineligible as PS covariates. Imputing such variables across arms would generate model-based pseudo-values without adding information for confounding control. For covariates included in the PS model, the proportion of missing values was small and was handled using categorical ‘missing’ levels.

The selection and assessment of the models were based on the Box-Cox test, the Hosmer-Lemeshow test, and the log-likelihood. Interactions between variables were included if their inclusion substantially improved the fit of the model to the data, as assessed by the Hosmer-Lemeshow test. Reweighted IPD were analyzed using the Cox model (with study group as the only covariate; robust variance estimators) and the Kaplan-Meier method.

#### 2.4.5 Adjusted comparison of OS: Cox model with grouping variable and confounders

A multivariable Cox model was planned to verify the results of the PS inverse probability or odds weighting model. The following potential covariates were considered in the model comparing dB to historical controls: time from diagnosis to the starting point, age at diagnosis, sex, MYCN status, and INSS stage.

#### 2.4.6 Technical note

A p-value of less than 0.05 was considered significant. Data were analyzed using StataNow 19.5SE (StataCorp., College Station, TX, USA) and OriginPro 2025b (OriginLab, Northampton, MA, USA).

## 3 Results

### 3.1 Patient characteristics

Significant differences were observed before weighting between patients who received dB and historical controls not treated with dB across all assessed baseline characteristics except sex. These included age at the starting point, age at NBL diagnosis, year of diagnosis, time from diagnosis to the starting point, MYCN status, and INSS stage at diagnosis (**Table 4**).

**Table 4:** Characteristics of the study population by arm (before and after PS reweighting).

Characteristic			Before PS reweighting			After PS reweighting			
			dB (N = 144)	Historical control (N = 82)	P-value	Characteristic	dB (N = 144, Σ(w) = 228.7)	Historical control (N = 82, Σ(w) = 203.8)	P-value
Age, years	Median (IQR)		7.96 (5.58 to 10.77)	6.60 (5.25 to 7.72)	**0.004**	Median	7.1	6.9	0.661
	Mean (SD)		8.64 (4.31)	7.01 (2.8)		Mean (95% CI)	8.0 (7.3 to 8.7)	7.7 (6.3 to 9.1)	
	n (%)	<2 years	1 (0.7%)	0 (0%)	**0.001**	%	0.5%	0.0%	**0.022**
		2 to 5 years	25 (17.4%)	17 (20.7%)			21.3%	20.5%	–
		5 to 10 years	70 (48.6%)	57 (69.5%)			52.2%	65.1%	–
		10 years and older	48 (33.3%)	8 (9.8%)			26.1%	14.3%	–
Age at diagnosis, years	Median (IQR)		4.04 (2.47 to 5.75)	3.74 (2.77 to 5.27)	0.788	Median	3.8	3.8	0.991
	Mean (SD)		4.51 (3.14)	4.19 (2.27)		Mean (95% CI)	4.4 (3.9 to 4.9)	4.4 (3.4 to 5.3)	
	n (%)	<2 years	25 (17.4%)	12 (14.6%)	0.448	%	18.2%	17.9%	0.977
		2 to 5 years	70 (48.6%)	47 (57.3%)			5.2%	4.6%	
		5 to 10 years	40 (27.8%)	21 (25.6%)			49.3%	51.3%	
		10 years and older	9 (6.3%)	2 (2.4%)			27.3%	26.2%	
Year of diagnosis	Median (IQR)		2009 (2008 to 2012)	2003.5 (2002 to 2006)	**By design**	Median	2010	2003	**By design**
	Mean (SD)		2009.5 (3.4)	2004 (2.8)	**(0.001)**	Mean (95% CI)	2010.1 (2009.5 to 2010.6)	2003.9 (2003.2 to 2004.5)	**(<0.001)**
Time from diagnosis, years	Median (IQR)		3.01 (2.29 to 5.29)	2.41 (2.02 to 2.91)	**0.001**	Median	2.7	2.5	0.338
	Mean (SD)		4.12 (2.75)	2.82 (1.3)		Mean (95% CI)	3.6 (3.3 to 4.0)	3.3 (2.7 to 3.9)	
	n (%)	1^st^ tercile (below 2.3 years)	39 (27.1%)	37 (45.1%)	**<0.001**	**%**	33.4%	37.7%	0.582
		2^nd^ tercile (2.3 to 3.7 years)	44 (30.6%)	31 (37.8%)			33.7%	33.3%	
		3^rd^ tercile (3.7 years and more)	61 (42.4%)	14 (17.1%)			32.9%	29.0%	
Sex	n (%)	Male	82 (56.9%)	55 (67.1%)	0.134	%	60.7%	59.7%	0.824
		Female	62 (43.1%)	27 (32.9%)			39.3%	40.3%	
MYCN status	n (%)	not amplified	117 (81.3%)	52 (63.4%)	**0.006**	%	73.4%	64.9%	0.164
		amplified	18 (12.5%)	24 (29.3%)			20.2%	26.7%	
		missing	9 (6.3%)	6 (7.3%)			6.5%	8.4%	
INSS stage at diagnosis	n (%)	4	108 (75%)	79 (96.3%)	**<0.001**	%	83.0%	87.6%	0.184
		Others ^A^ (1, 2A, 2B, 3, 4S, missing)	36 (25%)	3 (3.7%)			17.0%	12.4%	

^A^ combined due to the unequal distribution of each stage between arms. CI, confidence interval; INSS, International Neuroblastoma Staging System; IQR, interquartile range; MYCN, m**y**elo**c**ytomatosis related oncogene **n**euroblastoma; PS, propensity score; SD, standard deviation. Σ(w), sum of weights.p-values below 0.05 are indicated in bold.

No significant differences were found after PS reweighting between patients who received dB and historical controls across all assessed baseline characteristics except the year of diagnosis. These included age at starting point, age at NBL diagnosis, time from diagnosis to the starting point, sex, MYCN status, and INSS stage at diagnosis.

### 3.2 Unadjusted comparison

**Figure 1:**
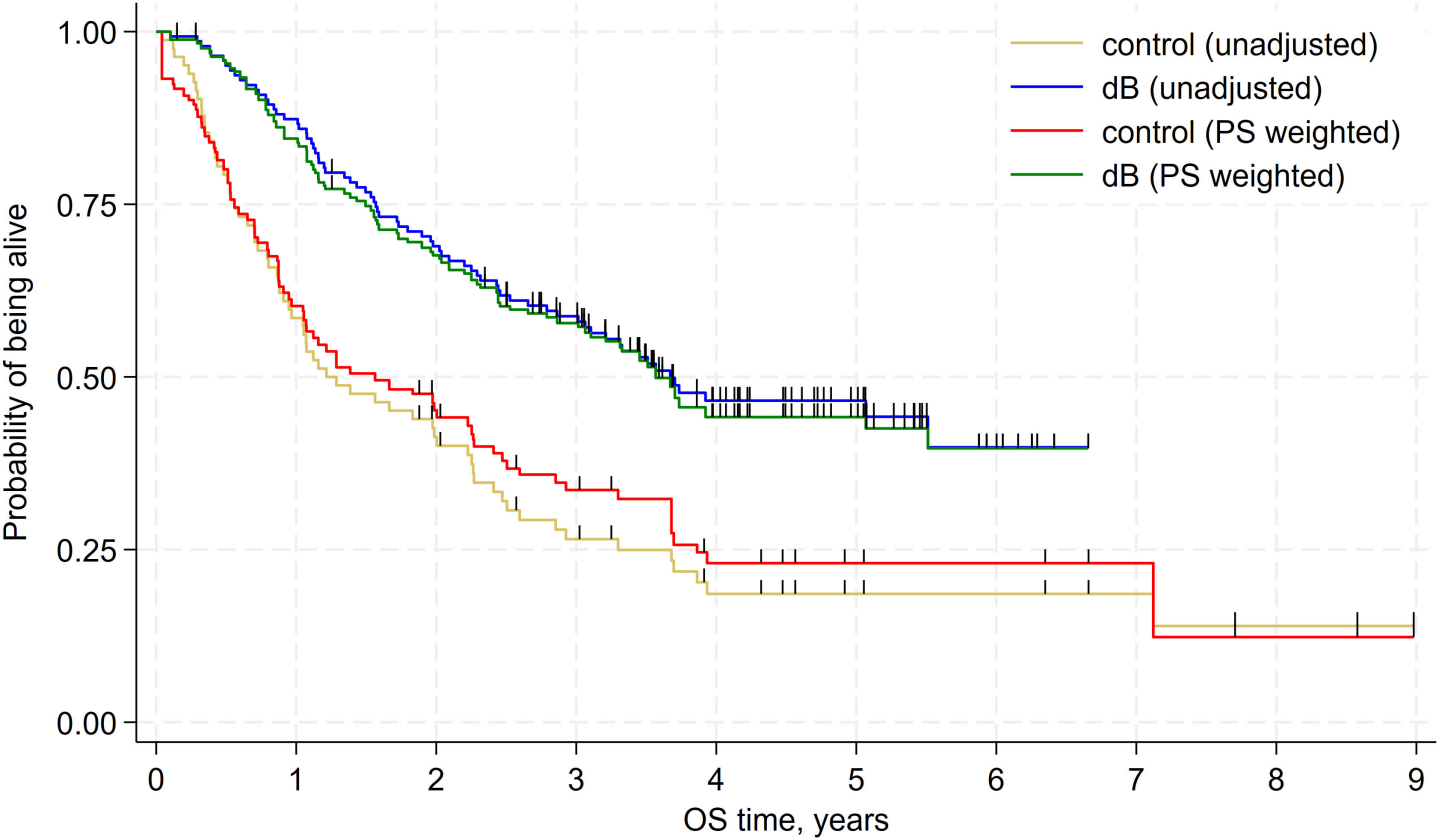
Kaplan-Meier plot showing the unadjusted and adjusted (propensity weighted) comparison of OS between dinutuximab beta (dB) and historical controls in relapsed neuroblastoma – base-case analysis.

**Figure 2:**
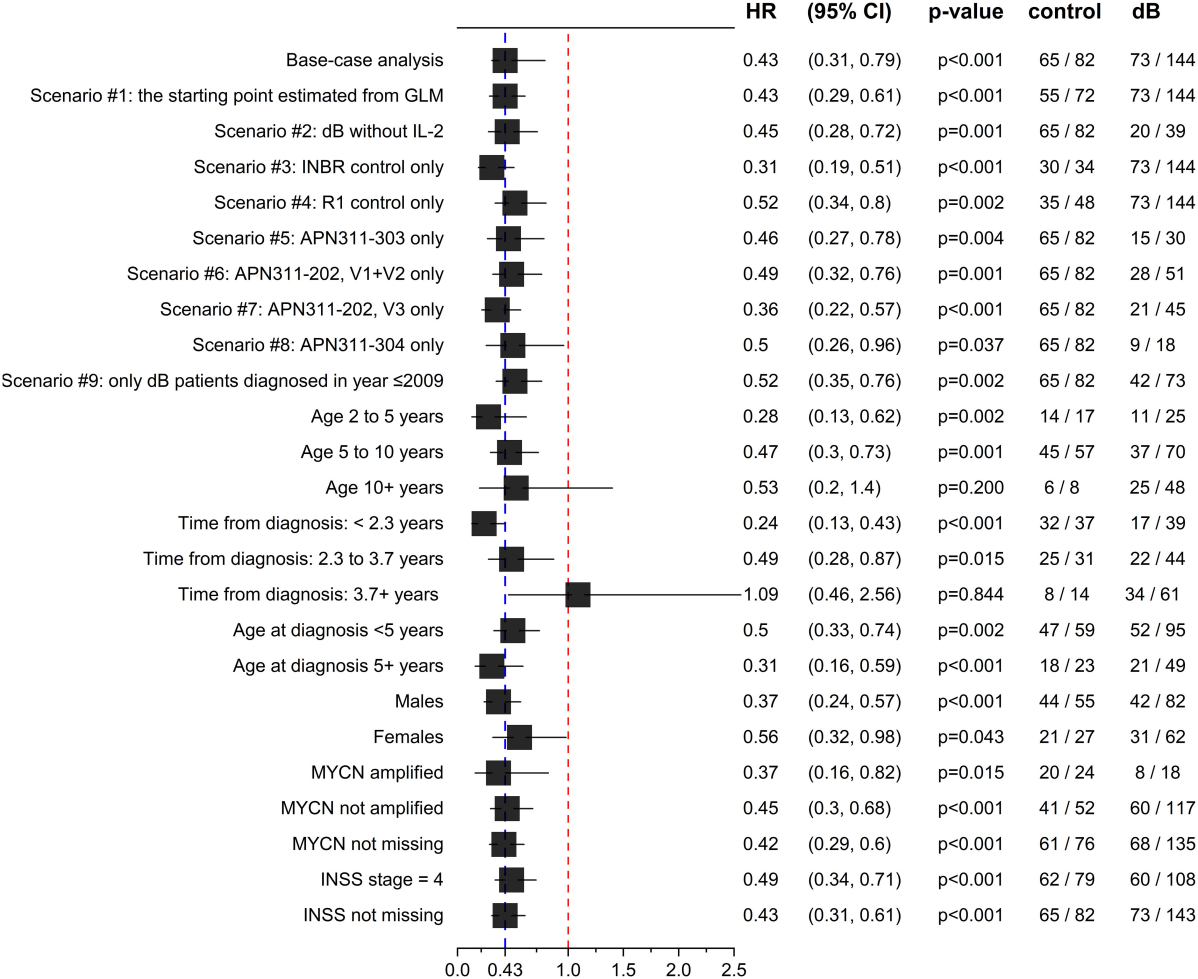
Sensitivity analyses of the unadjusted comparison of OS between dinutuximab beta (dB) and historical controls. HR (95% CI) and p-value from Cox models, with the number of events/patients for each arm.

The results of the base-case unadjusted indirect comparison (with data before PS reweighting) revealed that dB significantly prolonged OS compared to historical controls without dB treatment (hazard ratio [HR] = 0.43; 95% confidence interval [CI]: 0.31–0.79; p<0.001; **Table 5**). A clear separation of the Kaplan-Meier curves between the dB and historical control groups was evident from the first months of follow-up (**Figure 1**). The results of the unadjusted sensitivity analyses across various scenarios and subgroups were consistent with the base-case comparison, except the subgroup with age ≥10 years at the start (non-significant dB benefit) and time from diagnosis to the starting point equal or greater than 3.7 years (non-significant control benefit), both of which included <10 events in control arm (**Figure 2**, **Supplementary Table 1**).

**Table 5:** Unadjusted comparison of OS between dB and historical controls in relapsed neuroblastoma.

Parameter	Unadjusted comparison: dB vs historical controls
Log-rank test, p-value	**<0.001**
Cox model: HR (95% CI)	0.43 (0.31 to 0.79)
Cox model: p-value	**<0.001**

dB, dinutuximab beta; CI, confidence interval; HR, hazard ratio.p-values below 0.05 are indicated in bold.

### 3.3 Adjusted comparison: PS inverse probability or odds reweighting

The results of the base-case adjusted indirect comparison revealed that dB significantly prolonged OS compared to the historical control group not treated with dB (HR = 0.53; 95% CI: 0.35–0.79; p=0.002). A clear separation of the Kaplan-Meier curves between the dB and historical control groups was maintained after PS reweighting (**Figure 1**). Sensitivity analyses yielded results consistent with the base-case analysis (**Table 6**, **Supplementary Table 2**).

**Table 6:** Scenario analyses of the population-adjusted comparison of OS between dB and historical controls.

Scenario	Cox model: HR (95% CI), p-value
The starting point estimated from the GLM (starting point calculated using reliable method, but 10 patients excluded from the control arm, i.e. those who died before the new starting point)	0.55 (0.36–0.84), **0.006**
dB without IL-2 (the impact of which on treatment benefits is not supported by evidence)	0.48 (0.24–0.99), **0.047**
R1 control only (prospectively collected control arm only)	0.61 (0.38–0.97), **0.038**

p-values below 0.05 are indicated in bold.

### 3.4 Adjusted comparison: Cox model with confounders

The multivariable Cox model (**Supplementary Table 3**) indicated that dB treatment was associated with a 49% reduction in the HR of death compared to historical controls. The HR adjusted for patient characteristics (including sex, MYCN status, INSS, age at diagnosis, and time from diagnosis to the starting point) was estimated at 0.51 (95% CI: 0.35–0.73; p<0.001). This result was consistent with that obtained from the PS reweighting analysis, thereby supporting the validity of the PS-based findings.

## 4 Discussion

Despite the growing body of clinical and real-world evidence, head-to-head comparisons between dB (with or without IL-2) and no dB treatment in the maintenance setting for patients with relapsed NBL are lacking. This absence is primarily due to ethical constraints, as a randomized controlled trial by Yu et al. (2010) (23) demonstrated that the addition of an anti-GD2 antibody (dinutuximab), combined with granulocyte-macrophage colony-stimulating factor and IL-2, to isotretinoin following intensive multimodal therapy significantly improves survival outcomes in patients with HR-NBL. Thus, the objective of our analysis was to conduct an indirect comparison of the efficacy of dB in maintenance treatment versus no anti-GD2 treatment in the population of patients with relapsed NBL. The only feasible analysis was an unanchored indirect comparison using available IPD from dB studies (APN311-202 (18), APN311-304 (37) and APN311-303 (18)) and from historical cohorts of untreated patients, including the INBR control from Garaventa et al. (2009) (33) and the R1 control from the HR-NBL-1/SIOPEN study (34).

Newly available clinical study reports enabled the pooling of data from 144 patients treated with dB, with or without IL-2, and 82 historical control patients who did not receive immunotherapy, a uniquely large sample with extended follow-up, given the rarity of NBL (44). Considering that this population consists exclusively of patients with relapsed disease, recruiting large cohorts for prospective trials poses substantial challenges. In other studies investigating dB use in relapsed/refractory NBL, the number of enrolled patients did not exceed 70 (25). Moreover, previously published indirect comparisons of dB versus no dB treatment in the relapsed setting, including those originally conducted for the European Medicines Agency (18) (as reported in the European Public Assessment Report) encompassed significantly fewer patients. These comparisons were limited to studies such as APN311–303 and APN311-202 (N = 48 in both) (18), or the analysis by Mueller et al. (2018) (30), which included 29 patients on dB treatment versus 27 control patients from the AIEOP database.

In our study, harmonized inclusion criteria were applied across all study populations, enhancing the likelihood of selecting patients with comparable known and unknown characteristics from different data sources. A two-stage adjustment process (i.e., selection of appropriate patients based on the harmonized criteria, followed by adjustment for key baseline characteristics) enabled a reliable comparison. Significant differences were found in key baseline characteristics (i.e., age, age at NBL diagnosis, year of diagnosis, time from diagnosis to the starting point, MYCN status, and INSS stage at diagnosis) between patients from the dB arm and historical controls. However, these differences were well balanced after PS reweighing. The only baseline parameter that remained insufficiently balanced was year of diagnosis. This imbalance was expected, as the studies on dB use and those involving historical control cohorts were conducted using different designs and during different time periods, making alignment on year of diagnosis unfeasible. Almost half of dB patients (n=71, 49%) were diagnosed after 2009, while control patients were diagnosed between 1999 and 2009. Over the years, the standard of care may have improved (apart from better availability of anti-GD2 treatments) for patients diagnosed later. This advancement may be partially responsible for the observed improved OS in dB-treated patients, which constitutes a limitation of our analysis. However, the resulting bias is unlikely to be clinically meaningful, given the lack of effective treatments in the setting of multiple relapses. While chemoimmunotherapy with dB has been shown to improve survival outcomes (45–49), its wider adoption occurred after the completion of the studies included in this analysis.

Another limitation of this study is the difference in response to induction therapy between patients in the R1 control group and those in the INBR control group. In the R1 group, only patients who experienced relapse after achieving an initial complete response were included in this analysis (based on IPD availability), whereas the INBR group included all relapsed patients with any prior response (both partial and complete responses). In the dB studies patients with both complete and partial response were included. It is well established that individuals with a prior partial response generally have a poorer prognosis compared to those who achieved a complete response. Therefore, the inclusion of only those R1 patients, who were required to have achieved a CR, in the pooled analysis may have led to an underestimation of the survival benefit associated with dB. Furthermore, the limited availability of information on historical control patients prevented full adjustment for baseline differences between groups, such as prior treatment history, number of previous relapses or progressions, and subsequent treatments. Therefore, residual confounding cannot be excluded. We adjusted for the main post-relapse prognostic factors, but other clinically important variables (end-of-induction response, detailed histology, ALK status, tandem transplant was not done in that era at all type of transplant, number of frontline anti-GD2 cycles, chemotherapy backbone at relapse) were not available in a harmonized form. For several of these, robust evidence for an independent ‘carry-over’ effect on OS after relapse, beyond time to relapse, age, INSS and MYCN, is lacking, yet some residual confounding is likely.

The only assessed endpoint was OS, which is considered the gold standard for evaluating the clinical benefit of cancer therapy, particularly in the absence of cross-over. Overall survival is straightforward to measure and is not subject to investigator bias. However, treatment efficacy may be influenced by subsequent therapeutic interventions, specifically those administered after relapse, which can vary across clinical settings. Consequently, data necessary to balance patient populations based on post-progression treatments were not available (50).

In dB studies, OS was defined as the time from the initiation of dB treatment to death. On the other hand, for historical control patients, OS was originally calculated from the date of the last relapse. To correct for differences between dB studies and historical control groups in the base-case analysis, the starting point was estimated by adding 269 days (i.e., the median time between the first relapse and the start of dB and/or IL-2 treatment for patients from the APN311–202 study) to the date of relapse in the historical control groups. A limitation of this analysis is that it cannot be ruled out that some historical control patients may have received an anti-GD2 agent as part of other studies. If anti-GD2 therapy positively influences OS, then the inclusion of such patients in the control group would likely attenuate the observed difference in OS between the treatment and control groups.

Progression-free survival could not be defined consistently because dates and criteria for progression after relapse were incompletely and non-uniformly recorded in the historical cohorts. Consequently, overall survival from first relapse was used as the primary comparative endpoint.

The results of both unadjusted and adjusted indirect treatment comparisons revealed a significant improvement in OS with dB therapy, with or without IL-2, compared to historical controls not treated with dB. This risk of death was reduced by 57% in the unadjusted comparison and by 47% in the adjusted analysis. Additionally, 24 unadjusted and 3 adjusted sensitivity analyses were consistent with the primary findings and confirmed a significant survival benefit of dB over no immunotherapy. Sensitivity analyses examining the concomitant use of IL-2 indicated that the survival benefit of dB without IL-2 was comparable to that of dB with or without IL-2.

The results obtained in this analysis are consistent with previous indirect comparisons of dB versus historical controls, as presented in the European Public Assessment Report for dB (18). In that analysis, the proportion of patients remaining alive at 3 years was nearly twice as high in the dB-treated group (from the APN311–202 and APN311–303 studies) compared to historical controls (R1 (34) and Garaventa et al. (33)) who did not receive immunotherapy in the relapsed NBL population. Similar results were reported by Mueller et al. (2018) (30) for the APN311–303 study alone, in which the 3-year survival rate among patients with relapsed NBL treated with dB was twice as high as that of patients not treated with dB in the historical control cohort from the AEIOP database (54% vs 26%). As reported in the analysis by Wex et al. (2023), the comparison of dB plus IL-2 with the control cohort from the AEIOP database based on earlier data yielded a HR for OS of 0.43 (95% CI: 0.24–0.78); p=0.00542), while the HR of 0.56 (95% CI: 0.32–0.97; p=0.0376) was reported for comparison with the R1 control group (51).

A similarly beneficial effect on OS was demonstrated in primary studies evaluating anti-GD2 therapy in other subpopulations of patients with NBL, where immunotherapy was the primary intervention in the treatment group. In a meta-analysis by Khan et al. (2024) (52), dinutuximab was associated with a significant reduction in overall mortality, with a pooled relative risk of 0.41 (95% CI, 0.22–0.75; p = 0.004), and improved 5-year event-free survival among patients diagnosed with HR-NBL compared to alternative treatment modalities. Similarly, in the study by Ladenstein et al. (2020) (8), the 5-year event-free survival and OS rates for patients with HR-NBL who did not receive immunotherapy were 42% and 50%, respectively, whereas for those treated with dB, the rates were 57% and 64%, respectively (p<0.001) with the corresponding OS HR = 0.70 (95% CI: 0.54-0.91); p=0.0071 (53). Also, a retrospective observational study from Saudi Arabia (54) demonstrated that dB significantly improves survival outcomes in children with HR-NBL as compared with a control cohort, with a median OS of 46.88 months vs 15.91 months (p=0.001). Real World Evidence from the Netherlands Cancer Registry similarly confirmed efficacy of anti-GD2 therapy relative to non-immunotherapy control, adjusting for potential change in clinical practice, with HR = 0.37 (95% CI: 0.19-0.72); p <0.01 (55).

A factor that can potentially impact the magnitude of the treatment effect of dB in relapsed patients is prior exposure to dB or other anti-GD2 immunotherapies. Such an effect could be mediated through the presence of anti-drug antibodies or a reduced expression of GD2 antigen after relapse. Currently no clinical evidence is available allowing to confirm this effect, but anti-anti-GD2 antibodies were detected in patients treated with anti-GD2 immunotherapy with unknown clinical effect (14, 54), while high GD2 expression was reported to persist after relapse (56, 57). Complete or partial loss of GD2 expression was found in only 10-12% of neuroblastoma cells from bone marrow, with no clear relationship between GD2 expression and ADCC, suggesting that other mechanisms play more important role in developing resistance to treatment (58, 59). While it can be assumed that on multiple exposure, efficacy of anti-GD2 diminishes in some patients, our analysis demonstrates a high effect size, unlikely to be markedly reduced on re-treatment. Limited evidence suggests continued efficacy or anti-GD2 immunotherapies after prior exposure (48, 60, 61).

We lacked sufficiently detailed data to quantify prior anti-GD2 exposure in the DB cohort (some APN311 studies permitted enrolment of patients with prior anti-GD2 therapy provided no anti-DB antibodies were present). If prior anti-GD2 reduces the effectiveness of subsequent anti-GD2-based therapy at relapse, any such unmeasured exposure would be expected to bias our comparison toward the null, so the observed OS benefit associated with DB is likely conservative.

It should be noted that in all studies included in the indirect comparison, patients received dB via long-term infusion (LTI), administered over 10 days. LTI has been shown to provide a significantly greater survival benefit compared to short-term infusion, which is administered over 5 days, in patients with HR-NBL (37, 62). In addition, LTI is associated with a more favorable safety profile, including lower pain scores, reduced intravenous morphine use, and a lower incidence of grade ≥3 adverse events, which supports its feasibility in outpatient settings (30). These findings further support the use of LTI of dB, as recommended by SIOPEN, including in patients with relapsed/refractory NBL.

Although indirect comparisons do not represent the highest level of clinical evidence, they are commonly used in the assessment of treatments for reimbursement decisions. Regulatory authorities also accept evidence from single-arm studies using historical controls, as demonstrated by the recent approval of eflornithine by the U.S. Food and Drug Administration for first-line post-maintenance therapy in NBL (63). As confirmatory randomized studies comparing anti-GD2 therapies to treatment without immunotherapy are no longer considered for ethical reasons, this comparison constitutes best available comparative evidence informing clinical and reimbursement decisions.

## 5 Conclusion

Indirect comparisons inherently carry certain unavoidable methodological limitations due to differences in clinical experiences, which may affect the reliability of the results. Despite these limitations, and the inability to fully balance baseline characteristics across all potential prognostic factors, such as prior treatments or therapies received after progression, this study provides a robust comparison of OS in a context where conducting a parallel prospective trial of dB with a placebo control would not be ethically feasible. In this analysis, patients with relapsed NBL treated with dB were shown to have almost half lower risk of death compared to historical control patients. The magnitude of this treatment effect supports the efficacy of dB as maintenance therapy of NBL patients after relapse, even in the presence of potential biases. Unadjusted comparisons and multiple sensitivity analyses further confirmed these findings, reinforcing the strength of the conclusion.

## Data Availability

The original contributions presented in the study are included in the article/**Supplementary Material**. Further inquiries can be directed to the corresponding author.

## Supplementary material

Supplementary Table 1 Sensitivity analyses: scenario analyses and subgroup analyses of the unadjusted comparison of OS between dB and historical controls.

Supplementary Table 2 Results of the multivariable Cox model.

Supplementary Table 3 Scenario analyses of the population-adjusted comparison of OS between dB and historical controls.

## Glossary

13-cis-RA: 13-cis retinoic acid (isotretinoin)
AIEOP: Associazione Italiana di Ematologia e Oncologia Pediatrica
dB: dinutuximab beta
CI: confidence interval
CHO: Chinese hamster ovary cells
GD-2: disialoganglioside-2
HR: hazard ratio
HR-NBL: high-risk neuroblastoma
INBR: Italian Neuroblastoma Registry
INSS: International Neuroblastoma Staging System
NBL: neuroblastoma
IL-2: interleukin-2 (aldesleukin)
IPD: individual patient data
IQR: interquartile range
IU: international unit
LTI: long-term infusion
MAT: myeloablative therapy
MYCN: myelocytomatosis related oncogene neuroblastoma
OS: overall survival
PS: propensity score
SC: subcutaneous
SCT: stem cell transplantation
SD: standard deviation
