# Supplementary material for: Dinutuximab beta versus historical controls in the treatment of relapsed neuroblastoma: unadjusted and adjusted indirect comparisons
Source: Front Oncol. 2026 Jan 22;15:1736165. doi: 10.3389/fonc.2025.1736165 (PMC12872524; doi:10.3389/fonc.2025.1736165)
Supplement: Supplementary Table 1 — Sensitivity analyses: scenario analyses and subgroup analyses of the unadjusted comparison of OS between dB and historical controls. [file DataSheet1.pdf]

Supplement

SUPPLEMENTARY TABLE 1. Sensitivity analyses: scenario analyses and subgroup analyses of the unadjusted comparison of OS between dB and historical controls.

	Kaplan-Meier plot	Log-rank, p-value	Cox model: HR (95% CI), p-value
**0) Base-case analysis**	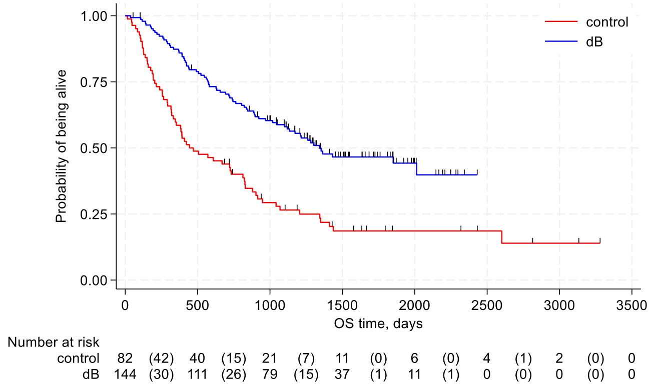	**<0.001**	0.43 (0.31 to 0.79), **<0.001**
**1) Scenario #1: the starting point was estimated from the generalized linear model (gamma distribution, log link, robust variance estimators)**	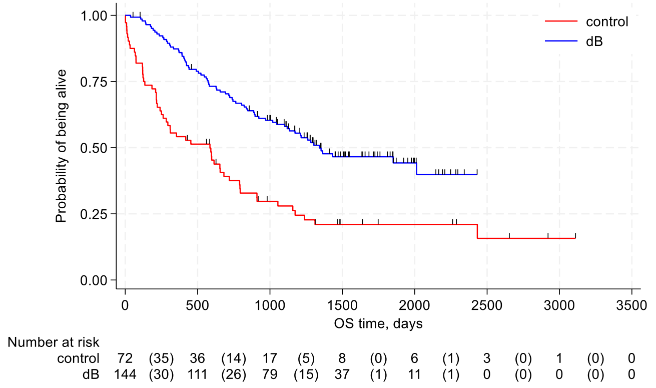	**<0.001**	0.43 (0.29 to 0.61), **<0.001**

	Kaplan-Meier plot	Log-rank, p-value	Cox model: HR (95% CI), p-value
**2) Scenario #2: dB without IL-2**	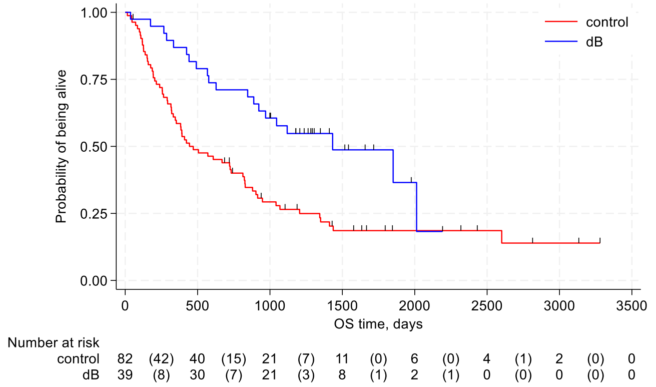	**0.0014**	0.45 (0.28 to 0.72), **0.001**
**3) Scenario #3: INBR control only**	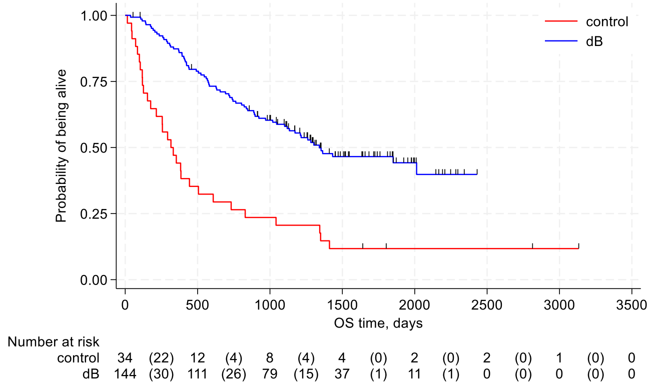	**<0.001**	0.31 (0.19 to 0.51), **<0.001**

	Kaplan-Meier plot	Log-rank, p-value	Cox model: HR (95% CI), p-value
**4) Scenario #4: R1 control only**	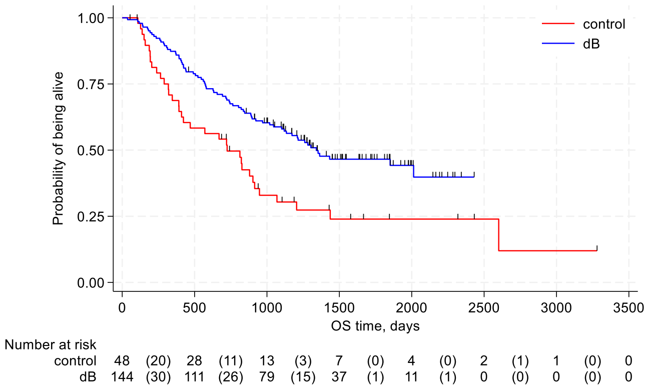	**0.002**	0.52 (0.34 to 0.80), **0.002**
**5) Scenario #5: APN311-303 only**	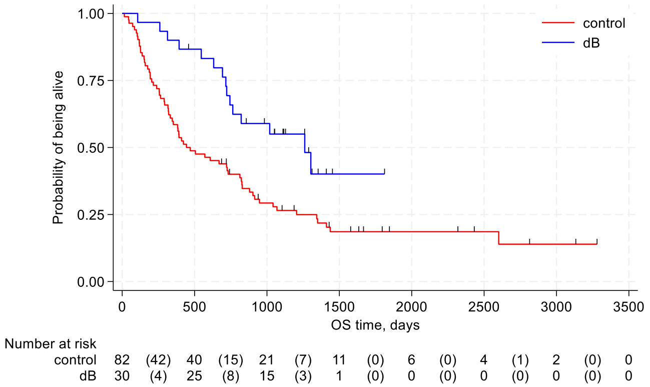	**0.006**	0.46 (0.27 to 0.78), **0.004**

	Kaplan-Meier plot	Log-rank, p-value	Cox model: HR (95% CI), p-value
**6) Scenario #6: APN311-202, V1+V2 only**	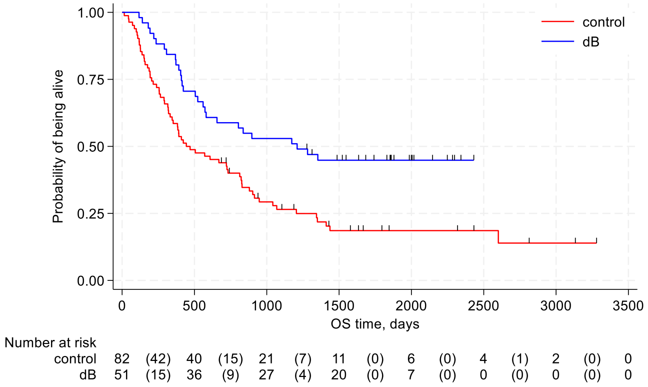	**0.0014**	0.49 (0.32 to 0.76), **0.001**
**7) Scenario #7: APN311-202, V3 only**	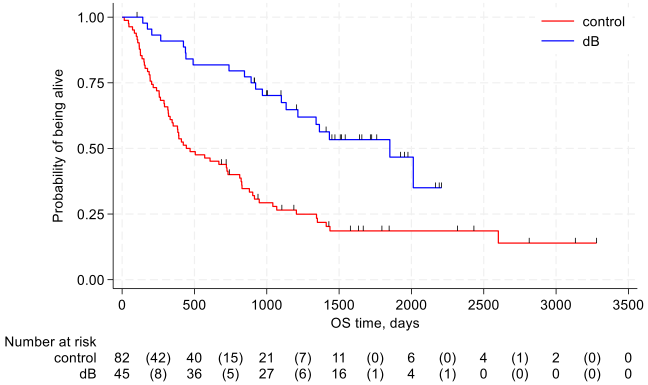	**<0.001**	0.36 (0.22 to 0.57), **<0.001**

	Kaplan-Meier plot	Log-rank, p-value	Cox model: HR (95% CI), p-value
**8) Scenario #8: APN311-304 only**	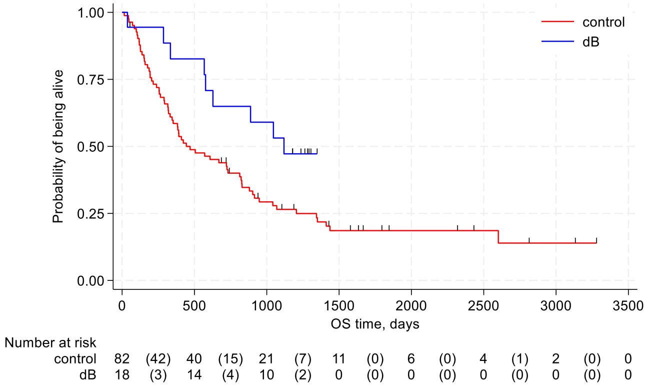	**0.047**	0.50 (0.26 to 0.96), **0.037**
**9) Scenario #9: only dB patients diagnosed in year ≤2009**	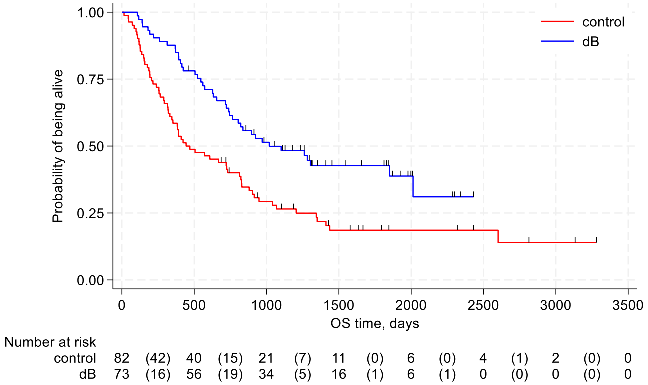	**0.001**	0.52 (0.35 to 0.76), **0.002**

<table>
<tr><th></th><th>Kaplan-Meier plot</th><th>Log-rank, p-value</th><th>Cox model: HR (95% CI), p-value</th></tr>
<tr><td>10) Age 2 to 5 years</td><td>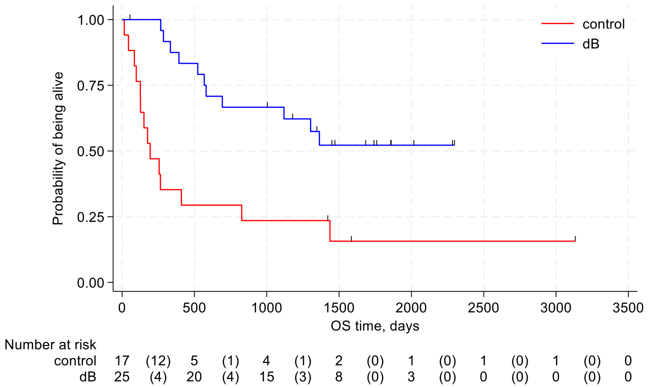
</td><td>0.001</td><td>0.28 (0.13 to 0.62), 0.002</td></tr>
<tr><td>11) Age 5 to 10 years</td><td>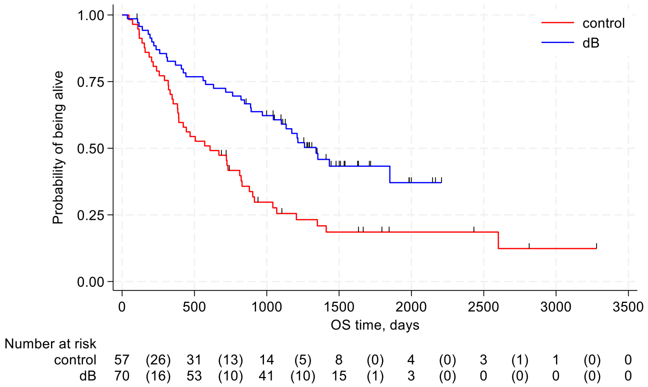
</td><td>0.001</td><td>0.47 (0.30 to 0.73), 0.001</td></tr>
</table>

<table>
<tr><th></th><th>Kaplan-Meier plot</th><th>Log-rank, p-value</th><th>Cox model: HR (95% CI), p-value</th></tr>
<tr><td>12) Age 10 years and older</td><td>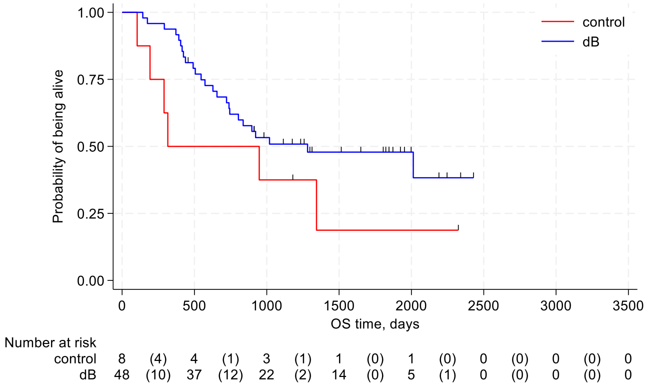
</td><td>0.154</td><td>0.53 (0.20 to 1.40), 0.200</td></tr>
<tr><td>13) Time from diagnosis: 1st tercile (below 2.3 years)</td><td>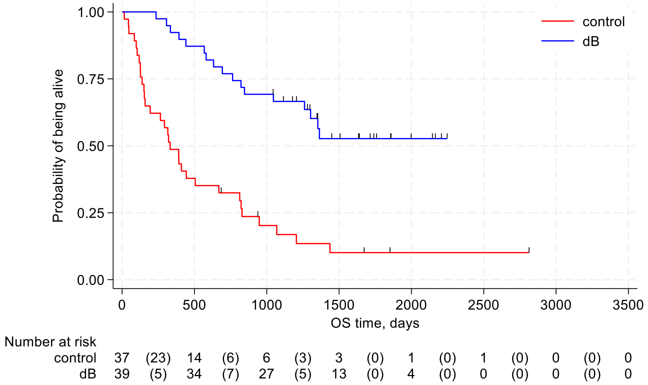
</td><td><0.001</td><td>0.24 (0.13 to 0.43), <0.001</td></tr>
</table>

	Kaplan-Meier plot	Log-rank, p-value	Cox model: HR (95% CI), p-value
**14) Time from diagnosis: 2nd tercile (2.3 to 3.7 years)**	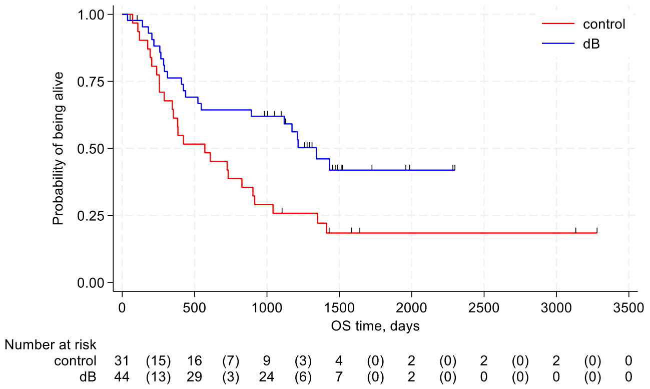	**0.013**	0.49 (0.28 to 0.87), **0.015**
**15) Time from diagnosis: 3rd tercile (3.7 years and more)**	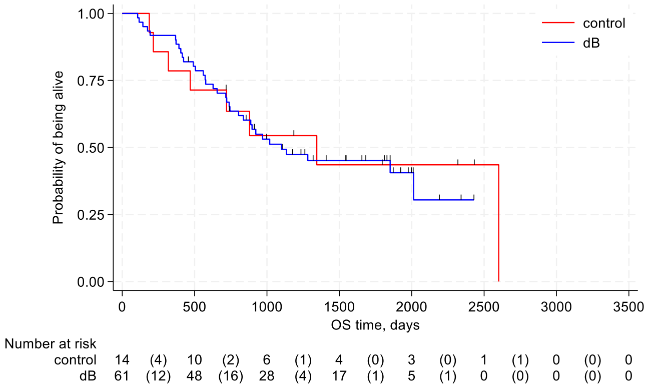	0.837	1.09 (0.46 to 2.56), 0.844

	Kaplan-Meier plot	Log-rank, p-value	Cox model: HR (95% CI), p-value
**16) Age at diagnosis <5 years**	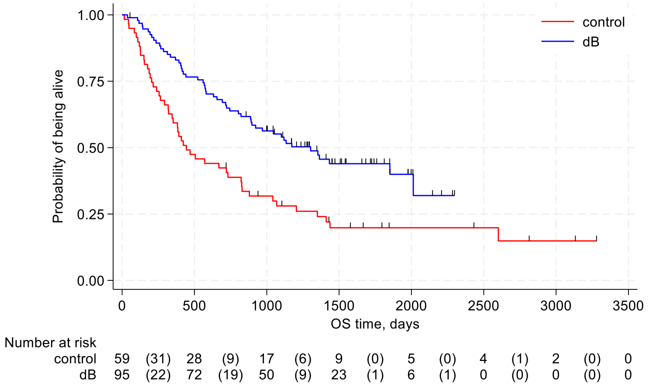	**0.001**	0.50 (0.33 to 0.74), **0.002**
**17) Age at diagnosis 5 years and older**	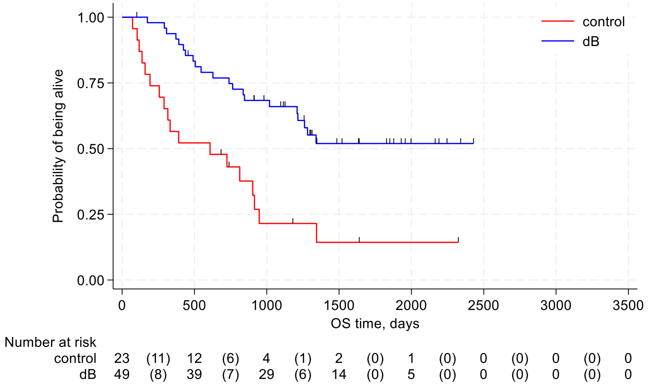	**<0.001**	0.31 (0.16 to 0.59), **<0.001**

	Kaplan-Meier plot	Log-rank, p-value	Cox model: HR (95% CI), p-value
**18) Males**	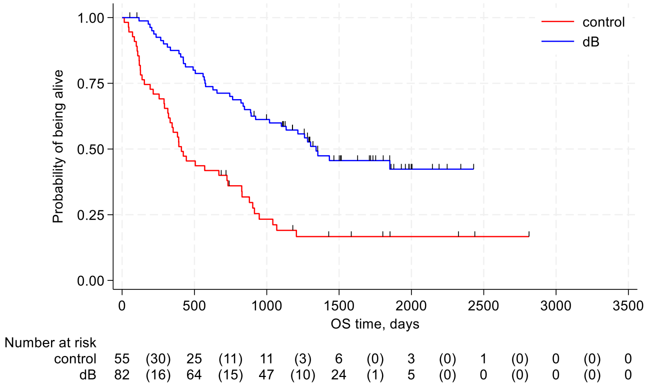	**<0.001**	0.37 (0.24 to 0.57), **<0.001**
**19) Females**	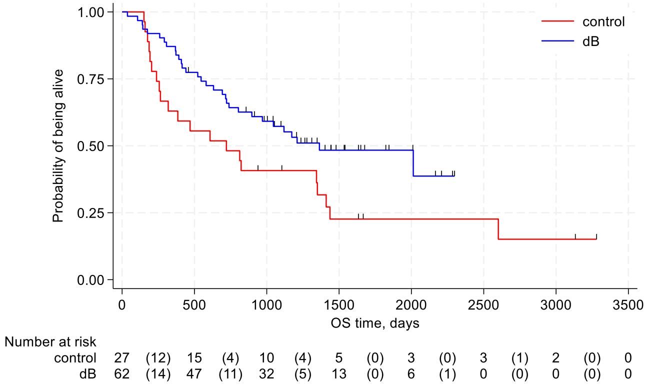	**0.041**	0.56 (0.32 to 0.98), **0.043**

	Kaplan-Meier plot	Log-rank, p-value	Cox model: HR (95% CI), p-value
**20) MYCN amplified**	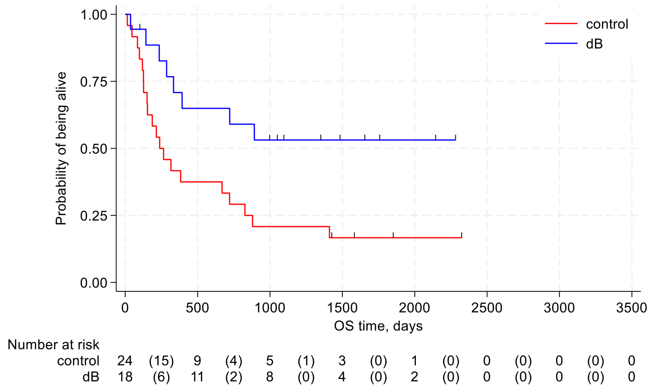	**0.013**	0.37 (0.16 to 0.82), **0.015**
**21) MYCN not amplified**	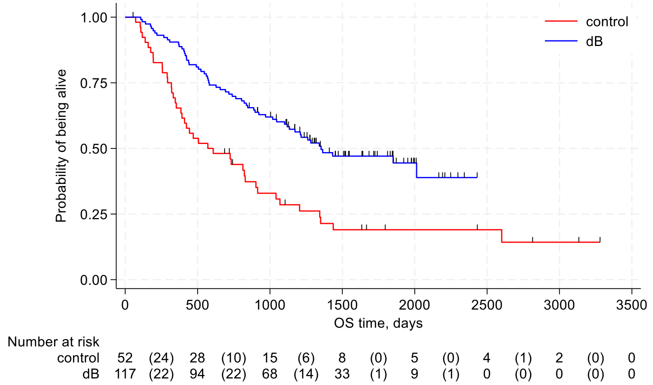	**<0.001**	0.45 (0.30 to 0.68), **<0.001**

<table>
<tr><th></th><th>Kaplan-Meier plot</th><th>Log-rank, p-value</th><th>Cox model: HR (95% CI), p-value</th></tr>
<tr><td>22) MYCN not missing</td><td>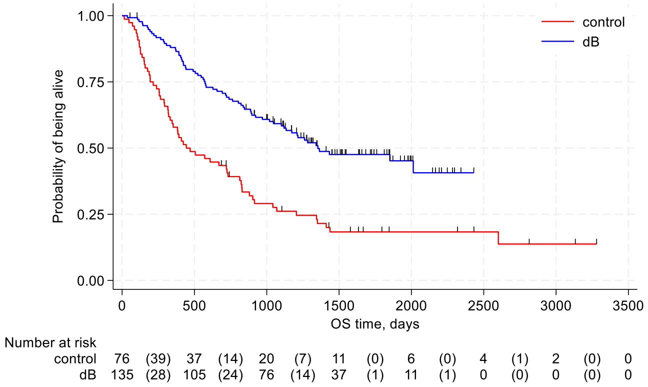
</td><td><0.001</td><td>0.42 (0.29 to 0.60), <0.001</td></tr>
<tr><td>23) INSS stage = 4</td><td>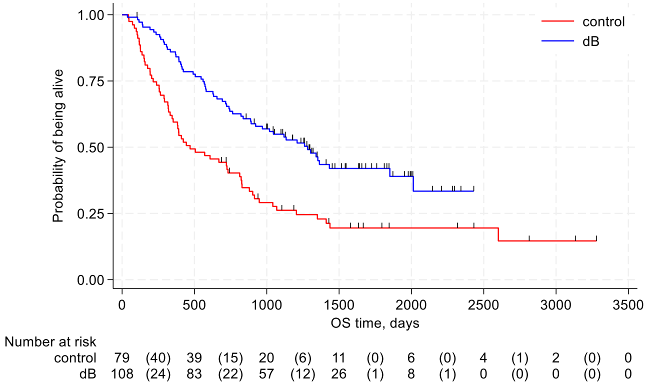
</td><td><0.001</td><td>0.49 (0.34 to 0.71), <0.001</td></tr>
</table>

	Kaplan-Meier plot	Log-rank, p-value	Cox model: HR (95% CI), p-value
**24) INSS stage not missing**	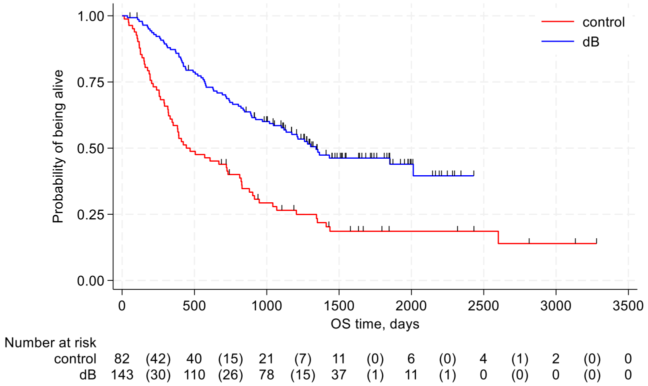	**<0.001**	0.43 (0.31 to 0.61), **<0.001**

SUPPLEMENTARY TABLE 2. Results of the multivariable Cox model.

```
Cox regression with Breslow method for ties

No. of subjects =     226                         Number of obs =     226
No. of failures =     138
Time at risk    = 218,098
                                                  Wald chi2(11) = 1391.53
Log pseudolikelihood = -661.91174                 Prob > chi2   =  0.0000
```

_t	Haz. ratio	Robust std. err.	z	P>|z|	[95% conf.	interval]
arm						
control	1	(base)				
dB	.5068344	.0940013	-3.66	0.000	.3523675	.7290148
sex						
male	1	(base)				
female	.8368167	.1533293	-0.97	0.331	.5843396	1.198382
mycn						
not amplified	1	(base)				
amplified	1.270273	.3247947	0.94	0.349	.7695812	2.096718
missing	1.387357	.4793676	0.95	0.343	.7048169	2.730866
inss						
1	1	(base)				
2B	1.746495	1.845512	0.53	0.598	.2201442	13.85567
3	3.108398	2.559862	1.38	0.168	.6187936	15.61448
4	3.001151	2.289708	1.44	0.150	.6727741	13.38771
4s	2.086828	2.153221	0.71	0.476	.2761874	15.76774
missing	1.78e-16	2.33e-16	-27.70	0.000	1.37e-17	2.32e-15
diag_age	.9669547	.0354907	-0.92	0.360	.8998374	1.039078
time_diag	.9722122	.0385159	-0.71	0.477	.8995788	1.05071

SUPPLEMENTARY TABLE 3. Scenario analyses of the population-adjusted comparison of OS between dB and historical controls.

	Kaplan-Meier plot	Cox model: HR (95% CI), p-value
**0) Base-case analysis**	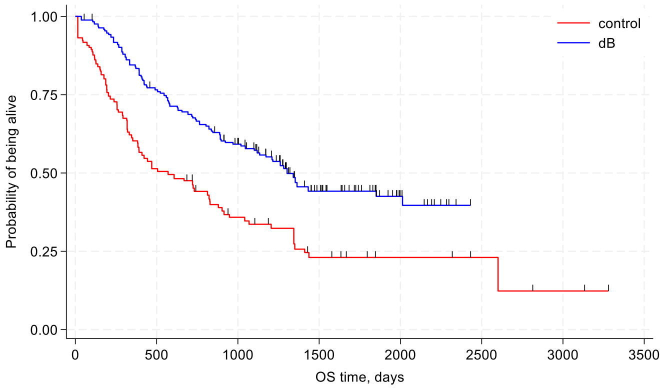	0.53 (0.35 to 0.79), **0.002**
**1) Scenario PS_1: the starting point estimated from the GLM**	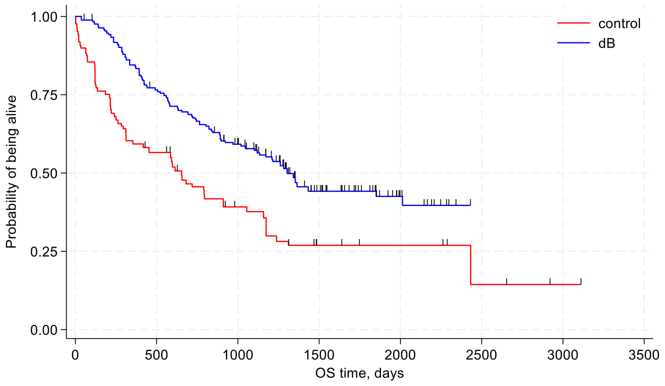	0.55 (0.36 to 0.84), **0.006** [starting point calculated using reliable method, but 10 patients excluded from the control arm (those who died before the new starting point)]
**2) Scenario PS_2: dB without IL-2**	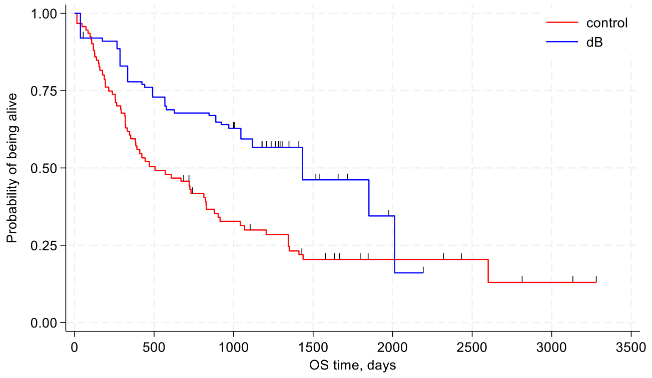	0.48 (0.24 to 0.99), **0.047** [without IL-2, the impact of which on treatment benefits is not supported by evidence]
**3) Scenario PS_3: R1 control only**	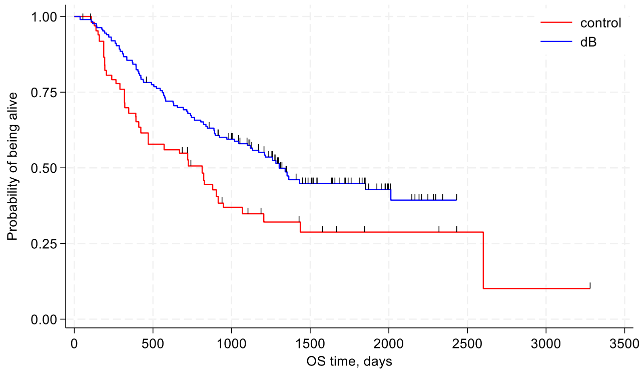	0.61 (0.38 to 0.97), **0.038** [vs prospectively collected control arm only]
